# Hydrogel‐embedded precision‐cut lung slices support ex vivo culture of in vivo‐induced premalignant lung lesions

**DOI:** 10.14814/phy2.70459

**Published:** 2025-07-12

**Authors:** Caroline Hauer, Rachel Blomberg, Kayla Sompel, Chelsea M. Magin, Meredith A. Tennis

**Affiliations:** ^1^ Division of Pulmonary Sciences and Critical Care Medicine, Department of Medicine University of Colorado Anschutz Medical Campus Aurora Colorado USA; ^2^ Department of Bioengineering University of Colorado Denver | Anschutz Aurora Colorado USA; ^3^ Department of Pediatrics University of Colorado Anschutz Medical Campus Aurora Colorado USA

**Keywords:** biomaterials, hydrogels, lung cancer, precision‐cut lung slice, premalignant lesion

## Abstract

Lung cancer is the leading cause of global cancer death, and prevention strategies are key to reducing mortality. Medical prevention of premalignant lesion (PML) progression may have a larger impact than treatment on mortality by targeting high‐risk populations and reducing their lung cancer risk. PMLs are difficult to study in humans but are easily accessible in murine preclinical carcinogenesis studies. Precision‐cut lung slices (PCLS) are underutilized as an ex vivo model for lung cancer studies due to limited culture time. Embedding PCLS within bioengineered hydrogels extends PCLS viability and functionality for up to 6 weeks. Here, we embedded PCLS with PMLs generated from urethane‐exposed mice in cell‐degradable and nondegradable hydrogels to study viability and responsiveness to PML interception over 6 weeks. PMLs in hydrogel‐embedded PCLS maintained viability, gene expression, and proliferation. Treatment of hydrogel‐embedded PCLS containing urethane‐induced PMLs with iloprost, a known lung cancer prevention agent, recapitulated in vivo gene expression and activity. Our studies also showed that iloprost reduced proliferation and PML size in PCLS embedded in degradable hydrogels. These results demonstrate that hydrogel‐embedded PCLS models support long‐term culture of in vivo generated PMLs. This modeling approach will improve preclinical studies of cancer biology and prevention across cancer types.

## 1 INTRODUCTION

Lung cancer is still the leading cause of cancer death in the United States among men and women and, while promising new therapies have been developed in recent decades, prevention remains the best strategy for reducing mortality from lung tumors (Siegel et al., 2023). Avoiding tobacco smoke exposure is the best behavioral prevention for many cancers, especially lung cancer, but in high‐risk populations, such as former smokers or cancer survivors, medical prevention approaches could have a significant impact on risk reduction. Historically, lung cancer prevention trials based on large population data sets, such as evaluation of β‐carotene, were not successful, while trials based on preclinical evidence, including the investigation of iloprost, demonstrated effects on intermediate endpoints like premalignant lesion (PML) histology (Keith et al., 2011; Kordiak et al., 2022). Accessing PMLs in human lung for mechanistic studies is challenging, reinforcing the critical role of preclinical studies in understanding the biology of PML progression and testing prevention agents (Le Magnen et al., 2016). Mouse studies relevant to human cancer, such as those incorporating tobacco carcinogens, provide access to multiple time points along the progression spectrum; however, these studies can be lengthy and costly (Bauer & Dwyer‐Nield, 2021; Sozio et al., 2021). Improvements in preclinical modeling are necessary to enhance research efficiency and to speed clinical translation of lung cancer prevention agents.

Precision‐cut lung slice (PCLS) models are used to study a variety of lung pathologies and treatments, including fibrosis, infections, and chronic obstructive pulmonary disease (COPD) (Liu et al., 2021; Meineke et al., 2022; Wronski et al., 2021). Ex vivo PCLS studies for lung cancer have been limited to a few publications investigating lung tumor biology and therapy, as well as a recent study from our group exploring PCLS as a model of lung carcinogenesis (Blomberg et al., 2023; Hu et al., 2023; Nagaraj et al., 2018; Narhi et al., 2018; Rubio et al., 2023; Sompel et al., 2023). PCLS are generated from lung tissue, most commonly mouse or human, by infusing tissue with agarose, slicing it, and culturing the slices floating in cell culture medium for experiments and analysis (Alsafadi et al., 2020). Cultured PCLS retain most cell types from the original tissue with intact structures and have expected responses to external stimuli. An added benefit of PCLS for animal studies is the ability to screen interventions ex vivo for efficacy prior to initiating in vivo studies, reducing animal number and burden. Other 3D models use single cell types or co‐cultures, but do not include multiple native cell types or maintain natural architecture (Urzi et al., 2023). The primary challenge of using PCLS for lung cancer studies is that the longevity of standard cultures is limited to around 1 week, as demonstrated in multiple previous studies (Koziol‐White et al., 2024; Lehmann et al., 2024). Short‐term PCLS cultures do not provide enough time for studies on lung cancer initiation, progression, or regression.

Recently, the Magin research group published an innovative approach to support PCLS ex vivo using tissue‐engineering strategies (Bailey et al., 2019). Lung tissue slices were embedded within engineered hydrogel biomaterials designed to extend maintenance of tissue architecture and viability for up to 3 weeks ex vivo (Bailey et al., 2019). In a subsequent study, we worked with a lung cancer research group to maintain the health of hydrogel‐embedded PCLS exposed to a cigarette‐smoke carcinogen for an unprecedented 6 weeks in culture (Blomberg et al., 2023). Carcinogen exposure promoted cellular proliferation and altered gene expression as expected based on other in vitro and in vivo studies (Blomberg et al., 2023). Here, our collaborative group presents the first study investigating the capacity of hydrogel‐embedded PCLS to maintain lung PMLs generated from an in vivo urethane adenocarcinoma carcinogenesis model. The present study explored PMLs in nondegradable hydrogel that was previously shown to best support lung tissue in long‐term culture and in a hydrogel degradable by matrix metalloproteinase‐9 (MMP‐9). MMP‐9 is an enzyme secreted by pulmonary and immune cells that is active during embryonic development for lung branching morphogenesis and angiogenesis, processes that may be aberrantly activated during lung cancer, and is associated with all stages and poor prognosis for lung cancer (Atkinson & Senior, 2003; Cabral‐Pacheco et al., 2020; Wei, 2023). In the present study, proliferation and gene expression within PMLs induced in vivo were maintained ex vivo for 6 weeks. Introduction of the prevention agent iloprost to hydrogel‐embedded PCLS changed gene expression, decreased proliferation, and reduced lesion area in agreement with previous preclinical and clinical studies (Keith et al., 2011; Miller et al., 2023; New et al., 2018; Sompel et al., 2022, 2023; Tennis et al., 2022). These changes were more consistently measured in the degradable hydrogel formulation indicating that it created a microenvironment that was more permissive to interception. Collectively, these results demonstrate feasibility of using hydrogel‐embedded PCLS to study the biology and interception of lung PMLs, that PCLS can be influenced by embedding hydrogels that mimic the tissue microenvironment, and suggest this novel model could extend the utility of PCLS for lung cancer prevention drug discovery and validation.

## 2 MATERIALS AND METHODS

### 2.1 Generation of mouse lung premalignant lesions

Wild type female A/J mice (8 weeks old, Jackson Laboratories) were housed in a pathogen‐free facility in the University of Colorado Anschutz Medical Campus Vivarium and fed AIN‐67 diet (#D10001, Research Diets, Inc.). Only female mice were used because males in the urethane model occasionally have increased aggression, and the risk of injury outweighed the benefit of males in this study. At 10 weeks old, mice were injected intraperitoneally with 100 μL of 1 mg urethane/gram (#943‐50 g, Sigma‐Aldrich) body weight dissolved in 0.9% saline vehicle or 100 μL 0.9% saline vehicle to induce PMLs associated with the development of adenocarcinoma (Sozio et al., 2021). Mice were weighed daily for 7 days after urethane injection and weekly for the remainder of the experiment, per standard protocols, and no unexpected weight changes were observed. 13 or 15 weeks after urethane exposure, mice were euthanized with carbon dioxide and exsanguination, and lungs were harvested for PCLS. Similar adenomas are present at 13 and 15 weeks, and any differences were not expected to affect study goals or endpoints. Animal numbers for urethane exposure were based on expected lung slice/punch yield, and punch number was determined by endpoint assay needs. All animal experiments and procedures were reviewed and approved by the University of Colorado Anschutz Medical Campus IACUC (Protocol #1085).

### 2.2 PCLS generation

Lungs were cleared with a cardiac perfusion of 10 mL sterile phosphate‐buffered saline (PBS; Gibco) through the right ventricle. Immediately after, the lungs were filled via tracheal perfusion with 1 mL of 1.5% low melting point agarose (#16‐520‐050, Invitrogen) dissolved in sterile 4‐(2‐hydroxyethyl)‐1‐piperazineethanesulfonic acid (HEPES) buffer. The whole mouse was placed on ice for 10 min before the lungs were extracted and placed in 1 mL of DMEM media with 0.1% Penicillin/Streptomycin/Fungizone (P/S/F) antibiotics/antimycotics (#SV3007901, Cytiva). Lung lobes were then cut into 500 mm slices with a vibratome (7000‐smz‐2, Campden Instruments) at 12 mm/s speed and standardized punches were created with a 4 mm biopsy punch (#NC9129052, Fisher Scientific). The punches were washed with media three times, with 30‐min incubations at 37°C between each wash to remove the agarose. Punches were cultured overnight in 24‐well plates before hydrogel embedding. Our methods are consistent with recent guidance on minimum reporting for PCLS preparation (Lehmann et al., 2024).

### 2.3 PEG‐Norbornene (PEG‐NB) synthesis

Norbornene‐conjugated eight‐arm 10 kg/mol PEG was generated as described previously (Caracena, Blomberg, Hewawasam, Riches, & Magin, 2022; Fairbanks et al., 2009). The purified product was characterized by 1H NMR (Bruker DPX‐400 FT NMR spectrometer, 300 MHz) and only the product that was at least 90% functionalized was used in experiments (Bailey et al., 2019; Blomberg et al., 2023).

### 2.4 Hydrogel formulations and rheology

Nondegradable hydrogel solutions consisted of PEG‐NB macromer (7 wt%), dithiothreitol (DTT) crosslinker (thiol:ene = 0.9; #AC165680050, Fisher Scientific), and the cellular adhesion peptides mimicking binding sites on fibronectin (CGRGDS; 0.1 mM), laminin (CGYIGSR; 0.2 mM), and collagen (CGFOGER; 0.1 mM) (custom synthesized by GL Biochem). Degradable hydrogel solutions consisted of PEG‐NB (7.5 wt%), an MMP‐9 degradable peptide crosslinker (Ac‐GCRD‐VPLSLYSG‐DRCG‐NH2; thiol:ene = 0.9; custom synthesized by GL Biochem), and the same cellular adhesion peptides (Patterson & Hubbell, 2010). For photopolymerization, 2.2 mM of the photoinitiator lithium phenyl‐2,4,6‐trimethylbenzoylphosphinate (LAP; #900889, Sigma Aldrich) was added. The elastic modulus of both formulations was determined by parallel plate rheology as previously described (Meyvis Tkl et al., 1999; Rubenstein & Colby, 2003).

### 2.5 Hydrogel embedding and hydrogel‐embedded PCLS culture

An 8‐mm diameter silicone mold facilitated hydrogel embedding as previously described (Bailey et al., 2019; Blomberg et al., 2023). First, 25 μL of the hydrogel precursor solution was added to the silicone mold and polymerized by exposure to 365 nm UV light at 10 mW/cm^2^ for 5 min. Hydrogel formulations were designed to be slightly off‐stoichiometry (thiol:ene = 0.9) to leave free reactive groups within this base layer after polymerization that enable the second hydrogel layer to covalently bond to the first. A 4‐mm PCLS punch was placed on the first hydrogel layer and covered with an additional 25 μL of hydrogel solution. The entire construct was exposed to 365 nm UV light at 10 mW/cm^2^ for 5 min to complete the final polymerization step, resulting in the PCLS punch being fully encapsulated in hydrogel on all sides. Hydrogel‐embedded PCLS were gently transferred to culture media in 24‐well plates and maintained at 37°C with 5.0% CO_2_ in DMEM:F12 (1:1; #11320033, Gibco) media with 0.1% fetal bovine serum (#A5256701, Gibco) and 0.1% 100X P/S/F antibiotics. Media were changed every 48 h. In chemoprevention experiments, hydrogel‐embedded PCLS cultures were treated with 10 μM iloprost (#18215, Cayman Chemical) or 9 mM methyl acetate vehicle control (#185325, Sigma Aldrich) every 48 h.

### 2.6 Presto blue metabolic activity assay

Hydrogel‐embedded PCLS were incubated in 500 μL of Presto blue reagent (#A13261, Invitrogen; 1:10 dilution in culture media) for 2 h at 37°C. Following incubation, 150 μL samples of Presto blue solution were transferred, in triplicate, to a clear 96‐well plate, and fluorescence intensity was measured at 520 nm excitation (Glomax plate reader; Promega). Fresh Presto blue solution was also read as a blank control, and average blank control fluorescence intensity values for each plate were subtracted from individual fluorescence intensity values. Weekly measurements were normalized to the average day zero reading for each group.

### 2.7 EdU incorporation assay

A 5‐ethynyl‐2′‐deoxyuridine (EdU) incorporation assay was performed according to the manufacturer's protocol (#C10337, Invitrogen). Hydrogel‐embedded PCLS were incubated for 16 h in complete culture media with 10 μM EdU. Samples were then fixed (4% PFA), permeabilized (0.5% Triton X‐100), and incubated in Click‐iT EdU reaction cocktail containing AlexaFluorazide for 30 min. Nuclei were then counterstained with 5 μM Hoechst 33342 (#H3570, Invitrogen). Samples were imaged on an upright epifluorescent microscope (Olympus BX63; Hamamatsu C11440 camera; cellSENS software RRID_SCR:014551) by acquiring a z‐stack at 10× magnification and then performing Wiener deconvolution. In Fiji (ImageJ), single channel z‐stacks were used to generate a maximum projection, thresholded to exclude background, and the number of nuclei was determined by counting particles. The number of EdU+ nuclei was divided by the number of total (Hoechst+) nuclei to calculate the percent proliferating cells.

### 2.8 RT‐qPCR

Three to five hydrogel‐embedded PCLS of the same experimental group were pooled together to form each sample, and RNA was extracted with the RNeasy Plus kit (#74134, Qiagen). qPCR was conducted on a CFX96 Touch (Bio‐Rad) using qPCR Prime PCR Assays (Bio‐Rad) C*es1* (qMmuCID0026391), *E‐cadherin (Cdh1)* (qMmuCED0044197), *Cox2 (Ptgs2)* (qMmuCED0047314), *Il6* (qMmuCID0005613), and *Vimentin (Vim) (*qMmuCED0046651) and the standard protocol for SsoAdvanced SYBR Green Master Mix (#1725274, Bio‐Rad). qPCR was conducted in triplicate. All gene expression data were normalized to the reference gene *Rps18*, and fold changes were calculated using the 2−ΔΔCt method.

### 2.9 Cryosectioning

Hydrogel‐embedded PCLS were fixed (4% paraformaldehyde; #50‐980‐487, Electron Microscopy Sciences) and quenched (100 mM glycine; #G46‐500, Fisher Scientific) for both steps at room temperature with rocking for 30 min. Samples were washed three times with PBS, excess hydrogel was trimmed from around the tissue, and then samples were placed in optimal cooling temperature (OCT; #23‐700‐571, Fisher Scientific) compound to perfuse at room temperature for 48 h. OCT‐infused samples were transferred to 10 × 10 × 5 mm cryomolds (#94‐4565‐1, Sakura), with 3–5 samples stacked into one mold, before being flash frozen by submersion in liquid nitrogen‐cooled 2‐methylbutane (#M32631, Sigma‐Aldrich). Blocks were stored at −80°C until cryosectioning on a Leica CM1850 cryostat. Frozen blocks were removed from the mold and mounted with one side pressed to the specimen disc, such that each cryosection would contain parallel cross‐sections of the stacked PCLS punches. Serial sections of 10 μm thickness were acquired at −20°C and stored at −80°C for further processing.

### 2.10 Immunofluorescence staining

Frozen sections were thawed to room temperature and equilibrated for 3 min in PBS before being circled by an ImmEdge hydrophobic pen (Vector Laboratories). Sections were blocked for 45 min in 5% BSA in PBS and then incubated with primary antibodies in 5% BSA overnight at 4°C. Primary antibodies were rabbit anti‐TTF1 (Abcam, #ab227652) and chicken anti‐KRT5 (BioLegend, #905901). Slides were washed three times in 0.1% Tween‐20 in PBS (PBST) before incubation with secondary antibodies (5 μg/mL; #A21437, #A23733, Invitrogen) in 5% BSA in PBS for 1 h at room temperature. After another three washes in PBST, sections were incubated in 300 mM DAPI for 15 min at room temperature and then washed a final time in PBS. Sections were mounted under a 24 × 50 mm coverslip in ProLong Gold antifade reagent (#P36930, Invitrogen) and allowed to cure overnight before imaging.

### 2.11 Peroxisome proliferator activated receptor gamma (PPARγ) activity assay

PPARγ activity was assessed using a PPARγ response element (PPRE) luciferase assay as described previously (Blomberg et al., 2023; Sompel et al., 2023). Immortalized Human Bronchial Epithelial Cells (CRL‐4051, ATCC) were transfected using TransIT‐X2 reagent (#MIR‐601, Mirus Bio). Conditions included 45 ng PPRE luciferase (Addgene plasmid #1015; gift from Bruce Speigelman) and 5 ng renilla control reporter vector (#E2261, Promega), mock luciferase, and empty vector. 24 h after transfection, 15 μL of media was collected from iloprost or vehicle‐treated PCLS and was added to HBEC cells. HBEC cells were incubated for an additional 24 h, and then luciferase activity was measured using the Dual‐Luciferase Reporter assay kit (#E2920, Promega) on a Glomax instrument (Promega). PPRE firefly activity was normalized to renilla control activity, and experimental groups were analyzed relative to vehicle controls.

### 2.12 Live cell imaging

For weekly imaging of live hydrogel‐embedded PCLS, samples were cultured in glass‐bottom 24‐well plates (#P24G‐0‐13‐F, MatTek Life Sciences). PCLS were stained in 250 μL of 5 μg/mL Hoechst 33342 and 2.5 μg/mL AlexFluor 647‐conjugated anti‐EpCAM (Biolegend, #118212) by incubating for 1 h at 37°C. Samples were washed three times in PBS and then allowed to rest before imaging for 1 h in 500 μL complete culture media. Immediately before imaging, 400 μL of media was removed, ensuring that the hydrogel‐embedded PCLS were resting on the glass bottom of the well. Imaging was performed on an inverted fluorescent microscope (BZ‐X100, Keyence) at 4× magnification, and then fresh media was added back to each well, and samples returned to long‐term culture. Images of the same lesions were captured weekly, and lesion size was quantified in Fiji (RRID:SCR_002285, ImageJ) by converting each dual‐channel image to 16‐bit grayscale, thresholding the positive signal, drawing a circular region of interest (ROI) around the lesion to exclude high signal coming from normal airways, and then finding the pixel area of the thresholded region. Area measurements from each week were normalized to the average lesion size for each group at time zero and presented as a percent.

### 2.13 Statistical analysis

Studies were conducted with a minimum of three biologic replicates and a standard of three technical replicates for the presto blue assay, qPCR, and the PPARγ activity assay. Technical replicates were averaged, and statistics were completed on biologic replicates. Data are reported as mean ± standard error of the mean (SEM). Experiments with two groups and a single independent variable were assessed by two‐tailed student's *t*‐test. Experiments with two independent variables were assessed by two‐way ANOVA with a Tukey's test for multiple comparisons. All statistical analyses were conducted in GraphPad Prism software (RRID:SCR_002798, GraphPad Software, Inc., San Diego, CA). Statistical significance is considered *p* ≤ 0.05.

## 3 RESULTS

### 3.1 Hydrogel embedding supported long‐term culture of hydrogel‐embedded PCLS with in vivo‐induced PMLs

**FIGURE 1:**
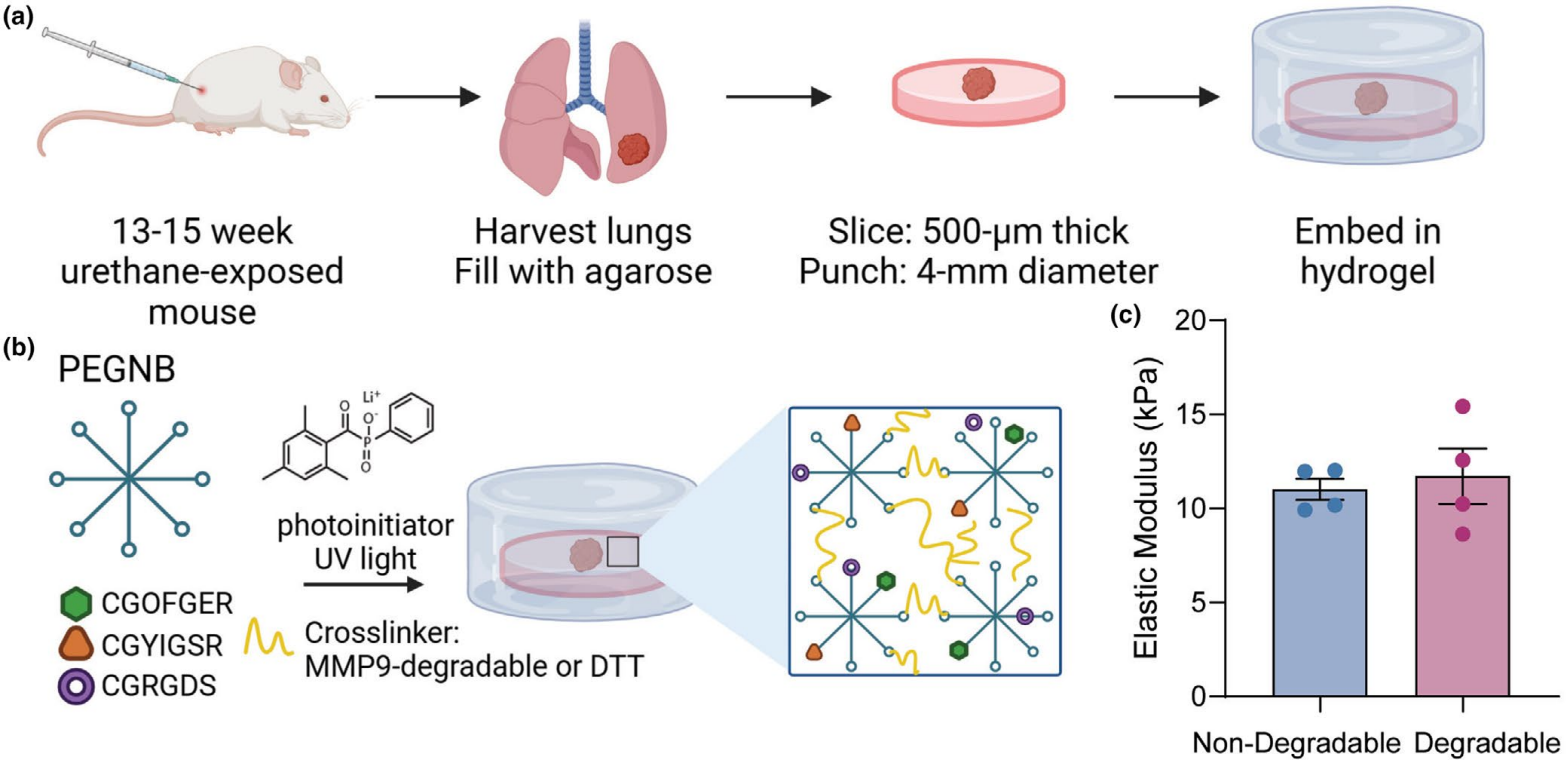
Generation of PML‐containing PCLS, embedding hydrogel composition, and rheology results. (a) The process of PCLS generation from urethane‐exposed mice included lung harvest, agarose filling of lungs, slicing filled lungs, and hydrogel embedding before culture. (b) Two hydrogel compositions included PEG‐NB, binding peptides (CGOFGER, CGYIGSR, and CGRGDS), a degradable or nondegradable crosslinker, and a photoinitiator for photopolymerization. (c) Rheology measurement of the elastic modulus (kPa) of nondegradable and degradable hydrogels (*N* = 4; no statistical differences, student's *t*‐test). Data are represented as mean ± SEM.

Lung tissue containing PMLs (urethane‐exposed) and vehicle controls (saline‐exposed) were generated by injecting A/J mice with urethane or saline, respectively, and harvesting lung tissue between 13 and 15 weeks (Figure 1a). Urethane is a tobacco carcinogen that consistently induces a KRAS mutation in mice, initiation of PMLs, and progression to carcinoma (Sozio et al., 2021). All PCLS from urethane‐exposed lung tissue contained a PML. After slicing, PCLS were embedded in nondegradable or degradable PEG‐NB hydrogel formulations containing peptide sequences mimicking collagen, laminin, and fibronectin (Figure 1b) and cultured at 5% CO_2_ and 37°C for 6 weeks. Rheology results showed the elastic modulus (stiffness) of nondegradable and degradable hydrogels was not statistically different and matched the stiffness previously determined to facilitate ex vivo induction of PMLs (Figure 1c) (Blomberg et al., 2023). Metabolic activity measurements were collected weekly using a Presto Blue assay.

**FIGURE 2:**
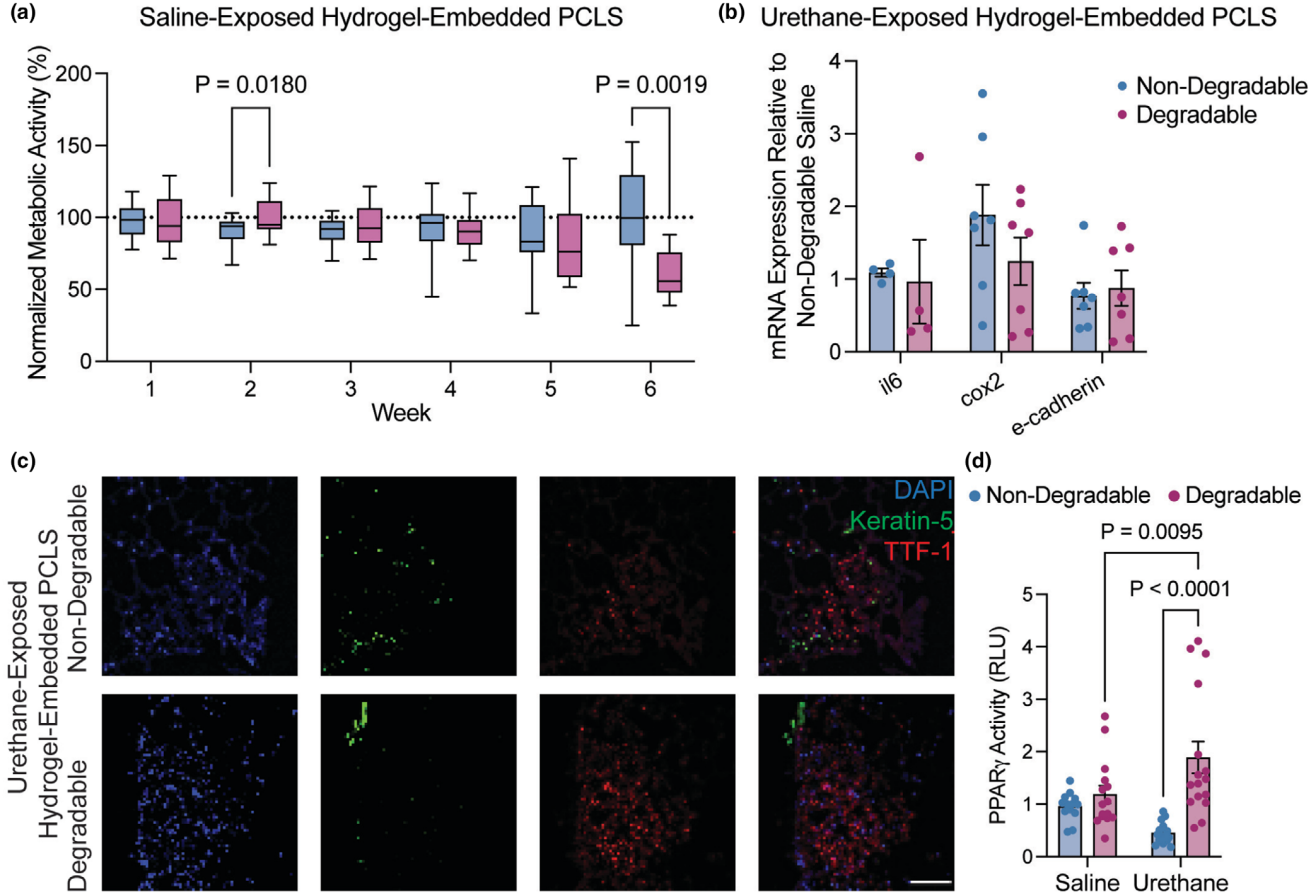
In vivo induced PMLs maintained metabolic activity and expression of lung cancer proteins over 6 weeks ex vivo. (a) Metabolic activity measurements normalized to initial readings showed cellular viability was maintained in saline‐exposed hydrogel‐embedded PCLS for 6 weeks. There was a statistically significant decrease in metabolic activity between nondegradable and degradable hydrogel samples at week 6. (*N* = 6) (b) Expression measured by qPCR for genes related to lung cancer premalignancy in urethane‐exposed samples was maintained and not statistically different between hydrogel compositions. (c) Representative fluorescence microscopy images of PML‐containing PCLS embedded in nondegradable and degradable hydrogels showing proteins associated with lung cancer premalignancy (Keratin 5 (green) and TTF‐1 (red)) counterstained with DAPI (blue) confirmed spatial arrangement and protein expression in PMLs are maintained over six weeks. Scale bar, 100 μM. (d) PPAR♥ activity in PCLS media at 6 weeks, measured by PPRE luciferase assay. Significance was determined by two‐way ANOVA and Tukey's test for multiple comparisons. Data are represented as mean ± SEM. Each dot represents a technical replicate.

Embedding PML‐containing PCLS in nondegradable or degradable hydrogels maintained viability of urethane‐exposed tissue with PMLs (Figure 2a) through 6 weeks as measured by metabolic activity. To compare hydrogel‐embedded PCLS containing PMLs to data from in vivo lung tissue containing PMLs, gene expression, immunofluorescence staining, and a PPRE luciferase assay for PPARγ activity were performed on hydrogel‐embedded PCLS. In mouse models, expression of inflammatory genes, such as *Cox2* (*Ptgs2*) and *Interleukin 6* (*Il6*), are increased, while epithelial genes, such as *E‐cadherin* (*Cdh1*), are reduced in PMLs and tissue from urethane‐exposed lungs (Narayan & Kumar, 2012; New et al., 2018; Smith et al., 2022; Tennis et al., 2022). Gene expression patterns in this study were not significant for *Il6*, *Cox2* (*Ptgs2*), and *E‐cadherin* (*Cdh1*) in urethane‐exposed, hydrogel‐embedded PCLS compared to saline‐exposed controls (Figure 2b). TTF‐1 identifies proliferating lesions in lung adenocarcinoma progression and differentiates them from squamous cell carcinoma lesions. In PCLS from in vivo urethane‐exposed lung tissue, TTF‐1 expression measured by immunofluorescence was present at 6 weeks in both nondegradable and degradable hydrogels (Figure 2c). In published studies of serum from the in vivo urethane model, PPARγ activity is reduced by urethane (Gu et al., 2023; Tennis et al., 2022). We found that at 6 weeks of culture, there was a significant increase in PPARγ activity in media from urethane‐exposed PCLS embedded in degradable hydrogels (*p* = 0.0095) compared to saline control (Figure 2d). PPARγ activity in media from PCLS embedded in degradable hydrogels was higher than activity in media from PCLS embedded in nondegradable hydrogels (*p* < 0.0001) (Figure 2d). These data suggest that lung tissue with PMLs from urethane‐exposed mice can be cultured for 6 weeks in hydrogel‐embedded PCLS with maintenance of viability and gene expression, but that specific activities may be influenced by the composition of the surrounding hydrogel.

### 3.2 Lung tissue with PMLs in hydrogel‐embedded PCLS recapitulated in vivo gene expression and signaling with chemoprevention

**FIGURE 3:**
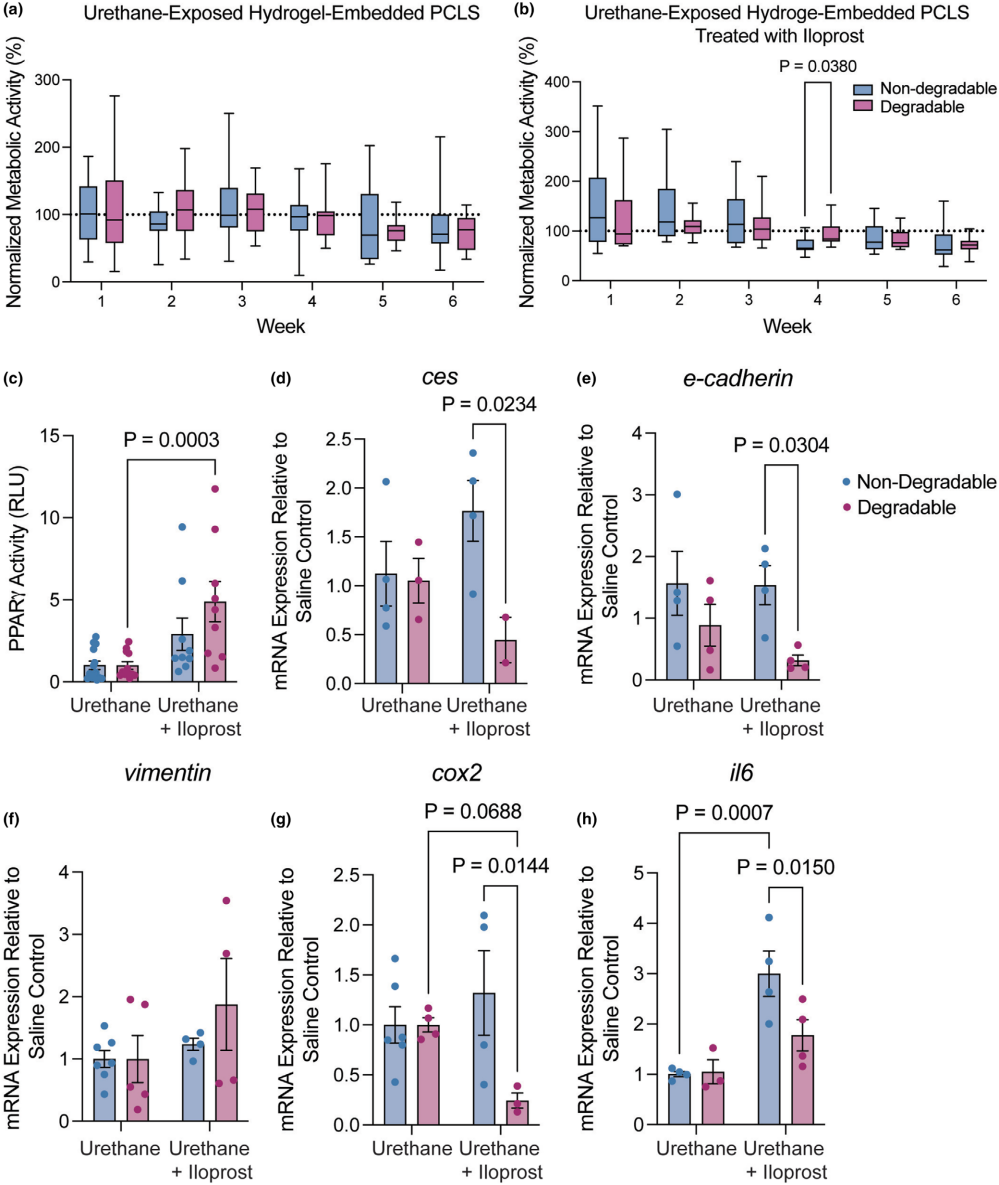
Hydrogel embedding supported tissue‐level responses to iloprost chemoprevention in PCLS with urethane‐induced PMLs. (a) Metabolic activity of urethane‐exposed hydrogel‐embedded PCLS treated with vehicle control. Results were normalized to initial readings and showed no significant decreases in PCLS viability over 6 weeks. (*N* = 6) (b) Metabolic activity of urethane‐exposed hydrogel‐embedded PCLS treated with iloprost. Results were normalized to initial readings and showed no significant decreases in PCLS viability over 6 weeks. (*N* = 6) (c) PPARγ activity in PCLS media, measured by PPRE luciferase assay increased in both hydrogels with iloprost treatment. qPCR measured expression of (d) *ces*, (e) *E‐cadherin* (*Cdh1*), (f) *Vim*, (g) *Cox2* (*Ptgs2*), and (h) *Il6*. Results were normalized to *Rps18* and presented relative to the hydrogel‐specific saline vehicle controls. Significance was determined by two‐way ANOVA and Tukey test for multiple comparisons. Data are represented as mean ± SEM. Each dot represents a technical replicate.

To compare the effects of a chemoprevention agent in hydrogel‐embedded PCLS to data from in vivo treated lung tissue, we measured gene expression and PPARγ activity. The lung cancer chemoprevention agent iloprost has been effective in numerous preclinical studies in mice, as well as in a phase II clinical trial of prevention in former smokers (Keith et al., 2011; Nemenoff et al., 2008; Tennis et al., 2022). Iloprost is a prostacyclin analogue that acts in part through PPARγ but likely has additional unknown targets related to its prevention activity (Nemenoff et al., 2008; Tennis, Van Scoyk, Heasley, et al., 2010). PCLS were generated from urethane‐exposed mouse lung tissue, embedded in nondegradable or degradable hydrogels, and then treated ex vivo with 10 μM iloprost or vehicle every 48 h for 6 weeks. Each PCLS from urethane‐exposed lungs contained a PML. Metabolic activity of the PCLS was maintained over 6 weeks of culture in nondegradable and degradable hydrogels for both vehicle controls (Figure 3a) and iloprost‐treated samples (Figure 3b). PPARγ activity increased in iloprost‐treated samples compared to vehicle controls in both nondegradable and degradable hydrogels with an increase in the degradable formulation (*p* = 0.0003) (Figure 3c).

Iloprost alters gene expression in vivo to mitigate the effects of carcinogen exposure, including increasing *E‐cadherin* (*Cdh1*) and *Ces1* and decreasing *Vim* and *Cox2* (*Ptgs2*) (Keith et al., 2010; New et al., 2018; Sompel et al., 2023). Here, iloprost did not significantly affect *ces* compared to vehicle in nondegradable hydrogel‐embedded PCLS or degradable hydrogel‐embedded PCLS (Figure 3d). There was a decrease in *ces* between nondegradable and degradable iloprost‐treated samples (*p* = 0.0234) (Figure 3d). Iloprost did not change *E‐cadherin* in non‐degradable hydrogels and decreased *E‐cadherin (Cdh1)* expression in degradable hydrogel‐embedded PCLS compared to nondegradable hydrogel‐embedded samples (*p* = 0.0304) (Figure 3e). *Vim* and *Cox2* (*Ptgs2*) expression did not significantly change in either hydrogel condition (Figure 3f,g). A significant decrease in *Cox2 (Ptgs2)* (*p* = 0.0144) was measured between nondegradable and degradable hydrogel formulations. In studies of lung and heart disease, iloprost either decreases or does not affect IL‐6; however, in short‐term PCLS experiments, we previously found increased IL‐6 with iloprost treatment (Sompel et al., 2023). *Il6* expression increased significantly with iloprost in nondegradable hydrogel compared to vehicle control (*p* = 0.0007) (Figure 3g). A significant decrease in *Il6* was measured with iloprost between nondegradable and degradable hydrogel microenvironments (*p* = 0.015). Observed changes in gene expression and signaling with ex vivo iloprost treatment of in vivo urethane‐exposed hydrogel‐embedded PCLS correlated with some previous results in whole lung tissue from in vivo iloprost treated mice, suggesting that iloprost may be active in hydrogel‐embedded PCLS (Miller et al., 2023; Sompel et al., 2022; Tennis et al., 2022). However, some observed differences indicate that hydrogel compositions may influence the microenvironment of PCLS and the activity of prevention agents.

### 3.3 Chemoprevention in hydrogel‐embedded PCLS reduces the proliferation and size of PMLs

**FIGURE 4:**
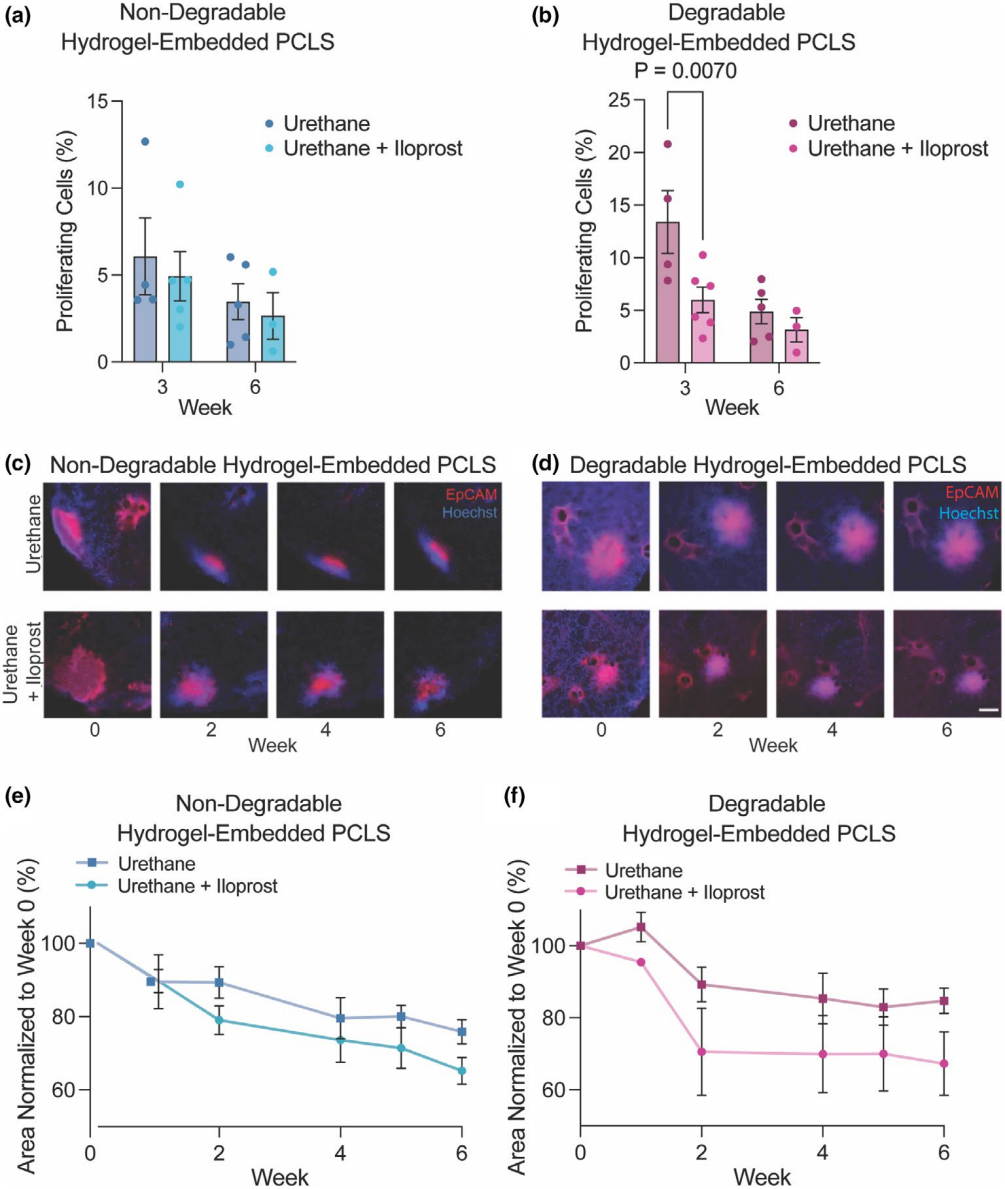
Iloprost treatment of urethane‐induced PMLs in hydrogel‐embedded PCLS reduced PML proliferation and size. Proliferation was measured by EdU and quantified as percent proliferating cells in PCLS embedded in (a) nondegradable hydrogel or (b) degradable hydrogel at 3 and 6 weeks. Proliferation was significantly decreased by iloprost treatment in degradable hydrogels at 3 weeks. PML size in PCLS was measured with live cell imaging using EpCAM and Hoescht staining every 2 weeks in PCLS embedded in (c) nondegradable or (d) degradable hydrogel. Scale bar is 100 μm. Quantification of live cell imaging as percent area of PML starting area in PCLS embedded in (e) nondegradable or (f) degradable hydrogel. Significance was measured by two‐way ANOVA and Tukey Test. All assays were conducted in triplicate. Data are represented as mean ± SEM.

To demonstrate the efficacy of a chemoprevention agent on PMLs in hydrogel‐embedded PCLS, proliferation and size of PMLs were measured during culture. Urethane‐induced lesions in vivo have increased proliferation (Fritz et al., 2014; Yano et al., 1997). In in vivo studies, iloprost reduced PML number, and in a human study, reduced PML grade (Keith et al., 2011; Nemenoff et al., 2008; Tennis et al., 2022). PCLS containing PMLs from urethane‐exposed mice or PCLS from saline‐exposed mice were generated, embedded in nondegradable or degradable hydrogel, and exposed to 10 μM iloprost or vehicle control every 48 h for 6 weeks. Analysis of proliferation by EdU at 3 and 6 weeks in nondegradable hydrogel‐embedded PCLS showed no significant differences in proliferation between timepoints or with iloprost treatment (Figure 4a). In contrast, proliferation was higher in degradable hydrogel‐embedded PCLS from urethane‐exposed lung tissue and significantly decreased with ex vivo iloprost treatment at week 3 (*p* = 0.007) (Figure 4b).

Real‐time live cell imaging with an anti‐EpCAM antibody monitored the size of PMLs over 6 weeks with measurements at 0, 1, 2, 4, 5, and 6 weeks (Figure 4c,d). Results for each timepoint were normalized to the original average size measured in each condition upon embedding (week 0). In PCLS embedded in the nondegradable hydrogel, PML area significantly decreased with iloprost treatment at weeks 2, 4, 5, and 6 (*p* = 0.01, 0.0006, 0.0002, and 0.0001, respectively) compared to time zero (Figure 4e). In the urethane‐exposed controls, a significant decrease in size was only observed at weeks 5 and 6 (*p* = 0.04 and 0.0070, respectively) compared to time zero (Figure 4e). The difference at 6 weeks in area between iloprost and control was about 10% but not statistically significant. In PCLS embedded in degradable hydrogel, the area of PMLs in urethane‐exposed PCLS increased at week one and, while PMLs decreased in area after this timepoint, these PMLs remained the largest over the 6 weeks of culture (Figure 4f). In degradable hydrogel, significant differences in area compared to time zero were not observed in the urethane‐only PMLs, while iloprost‐treated PMLs had significant decreases in area at weeks 4, 5, and 6 (*p* = 0.05, 0.05, 0.03, respectively) compared to time zero. The area of iloprost‐treated PMLs in the degradable hydrogel decreased at a faster rate than the urethane‐exposed, with a difference of about 20% at 6 weeks that was not significant. Overall, these results suggest that treatment with iloprost reduces proliferation and PML area in hydrogel‐embedded PCLS and that degradable hydrogels better maintained these characteristics of premalignancy and response to chemoprevention over time.

## 4 DISCUSSION

The results presented here are the first to demonstrate that lung PMLs induced in vivo by urethane can be maintained in hydrogel‐embedded PCLS for 6 weeks. Gene expression and signaling activity in hydrogel‐embedded PCLS cultured for 6 weeks recapitulated some previous results from in vivo lung models (New et al., 2018; Sompel et al., 2022; Tennis et al., 2022). Ex vivo prevention agent treatment of PMLs in hydrogel‐embedded PCLS mimicked some characteristics of in vivo treatments, with changes in gene expression, signaling activity, proliferation, and PML size associated with treatment (New et al., 2018; Sompel et al., 2022; Tennis et al., 2022). These results provide proof‐of‐concept data for the feasibility of hydrogel‐embedded PCLS to support extended study of PMLs and prevention ex vivo. This new model could accelerate our ability to discover and validate new chemoprevention agents.

Human lung cancer progression is associated with the deposition of dense extracellular matrix and increases in tissue stiffness, which supports epithelial and tumor cell proliferation (Kandice et al., 2009; Matthew et al., 2005; Paolo et al., 2008). In agreement with this in vivo phenomenon, we previously found that a stiff hydrogel incorporating the collagen‐derived peptide GFOGER best supported the initiation of carcinogenesis by vinyl carbamate (a metabolite of urethane) in hydrogel‐embedded mouse PCLS (Blomberg et al., 2023). Here, we build on this work by using stiff hydrogels containing the GFOGER sequence and interrogated whether changes in the hydrogel degradability might alter PML activity or growth. Nondegradable hydrogels containing the synthetic crosslinker DTT were compared to a peptide crosslinker in the degradable hydrogel that can be acted upon by the enzyme MMP‐9 that is secreted by various cell types in PCLS. Increased MMP‐9 expression is associated with all stages of lung cancer and poor prognosis, but has also been detected in mild squamous dysplasia, where cells expressing MMP‐9 moved through disrupted basal membranes with colocalized collagen and MMP‐9 (Galateau‐Salle et al., 2000; Wei, 2023).

One goal of these experiments was to determine if the ability to degrade the hydrogel environment through MMP‐9 activity would lead to effects on PMLs or response to treatment. PCLS embedded in degradable hydrogel and treated with iloprost had a decrease in proliferating cells at 3 weeks and reduced PML area over 6 weeks. PCLS embedded in nondegradable hydrogel did not have significant changes in proliferation or PML area with iloprost treatment. These phenotypic differences suggest the ability of PCLS to degrade surrounding hydrogel may lead to a microenvironment for PMLs that better replicates the in vivo response to iloprost treatment. The value of degradable over nondegradable hydrogel for PCLS is also supported by the observed increases of PPARγ activity with iloprost treatment. In one exception to the agreement with previous in vivo studies, PPARγ activity increased in degradable hydrogel‐embedded PCLS with urethane‐induced PMLs, as compared to saline controls, which may be due to interactions between PPARγ signaling and MMP‐9 itself. Inhibition of MMP‐9 occurs with PPARγ activation in lung tumor cell lines, a pathway that may be activated by increased availability of MMP‐9‐degradable material within the embedding hydrogel (Li et al., 2014; Magenta et al., 2008; Tennis, Van Scoyk, Freeman, et al., 2010). Increased activity of PPARγ in MMP‐9 degradable hydrogel‐embedded PCLS correlates with observed differences in proliferation and PML size with iloprost treatment in degradable hydrogels, as PPARγ activity is critical for effects of iloprost on lung cells (Nemenoff et al., 2008; Tennis, Van Scoyk, Heasley, et al., 2010). PCLS embedded in nondegradable hydrogel had few significantly different changes with iloprost, suggesting that while nondegradable hydrogel supports viability of PMLs, it may not be sufficient to generate expected phenotypes with treatment. These findings hint at an interplay between the extracellular microenvironment and effectiveness of chemoprevention agents that should be studied further in future work. Hydrogel embedding provides a platform for better understanding this relationship between extracellular matrix (ECM) components and chemoprevention efficacy. For example, future studies using this platform could focus on the mechanisms underlying the interactions observed here between MMP‐degradable microenvironments and PPARγ activity. Alternatively, the MMP9‐degradable crosslinker could be replaced with a peptide degradable by other MMPs or even with decellularized ECM from healthy and/or premalignant lung tissue to further elucidate the pathways responsible for the differing results observed here (Caracena, Blomberg, Hewawasam, Fry, et al., 2022; Hewawasam et al., 2023; Lutolf et al., 2003; Petrou et al., 2020; Saleh et al., 2022).

Chemical carcinogenesis models of lung adenocarcinoma in mice employ a range of carcinogens, but all use large numbers of mice and have long tumor latency (Bauer & Dwyer‐Nield, 2021; Sozio et al., 2021). Testing prevention agents in mouse lung adenocarcinoma carcinogen models requires even more animals to observe limited effect sizes (Chen et al., 2024; Kassie et al., 2022; Nana‐Sinkam et al., 2007; Nemenoff et al., 2008; Tennis et al., 2022). Despite challenges, preclinical studies are critical for advancing precision prevention approaches (Le Magnen et al., 2016). The hydrogel‐embedded PCLS model presented here could reduce the number of animals required for preclinical prevention agent studies by supporting high‐throughput ex vivo experiments for hypothesis refinement or agent screening prior to full in vivo studies. This model also allows investigations into stages and mechanisms of lung PML development that are not accessible in humans and offers more opportunities for manipulation than in vivo models (Abdull Razis, Bagatta, et al., 2011; Hu et al., 2023; Rubio et al., 2023). Previous lung cancer PCLS studies measured response to agents in tumor or normal tissue for periods of several hours to several days, but none studied PMLs or used long‐term culture (Abdull Razis, Iori, & Ioannides, 2011; Dong et al., 2011; Junk et al., 2021; Karger et al., 2023; Narhi et al., 2018; Prades‐Sagarra et al., 2024). While other three‐dimensional modeling systems may be useful to answer specific questions related to lung cancer, these models also have important limitations for lung premalignancy and prevention research. For example, patient‐derived tumor organoids may be an effective tool for identifying tumor‐specific treatments, but these cultures require a large number of initial cells, lack resident immune cells, and normal airway cells often overgrow the tumor cells (Dijkstra et al., 2020). Microfluidic lung‐on‐a‐chip models can recapitulate physiologic and mechanical functions that affect lung cancer development, but disrupt the native environment of tumor cells (Shukla et al., 2018; Xu et al., 2013). The complete tissue architecture and maintenance of many biologic processes in the hydrogel‐embedded PCLS model are critical for studying tissue‐level mechanisms, and this expansion of available preclinical models will support critical contributions to lung cancer research.

Our study generated PCLS from one in vivo time point; additional time points could be used in future studies to assess how progressive stages can be supported by hydrogel‐embedded PCLS, further expanding the application of the model. We previously demonstrated the presence of macrophages and T‐cells in hydrogel‐embedded PCLS at 6 weeks (Blomberg et al., 2023; Sompel et al., 2023). Comprehensive characterization of the presence or activity of immune cells throughout the 6‐week culture will support the adoption of this model to investigate immune mechanisms and test immunoprevention agents. This study focused on adenomatous PMLs induced by urethane; however, the model could also be applied to squamous cell carcinoma (SCC) by using the n‐nitroso‐tris‐chloroethylurea (NTCU) mouse model to generate PCLS (Dwyer‐Nield et al., 2020; Riolobos et al., 2019). We have explored using human tissue in hydrogel‐embedded PCLS for initiating PMLs ex vivo, but accessing human PMLs in tissue suitable for long‐term PCLS is challenging (Blomberg et al., 2023; Lehmann et al., 2024). We continue to investigate the possibilities for human‐relevant hydrogel‐embedded PCLS, including designing hydrogels to mimic human microenvironment conditions in mouse PCLS.

A hydrogel‐embedded PCLS model that allows long‐term culture of PMLs is an exciting development in preclinical modeling of cancer that is expected to have a significant impact on PML biology and prevention agent studies. Future studies focused on bioengineering aspects could include modifications of hydrogel composition to mimic different parts of the PML microenvironment. Future studies focused on lung cancer could investigate comorbidities with hydrogel‐embedded PCLS from COPD or fibrosis mouse models that are exposed to lung cancer carcinogens ex vivo. Humans are often exposed to multiple carcinogens, but in vivo studies of combined carcinogens are challenging, so hydrogel‐embedded PCLS could be used to explore biology with multiple exposures. While the focus of this study was on PMLs and prevention, this foundational hydrogel‐embedded PCLS model could also be used to study solid tumors and targeted therapies across cancer types, broadening the potential impact on cancer research.

## AUTHOR CONTRIBUTIONS

C. Hauer and K. Sompel were involved in investigation and writing – original draft. R. Blomberg was involved in investigation, methodology, formal analysis, writing – original draft, writing – review and editing. C.M. Magin was involved in conceptualization, funding acquisition, project administration, data curation, resources, supervision, and writing – review and editing. M.A. Tennis was involved in conceptualization, funding acquisition, project administration, supervision, writing – original draft, and writing – review and editing.

## FUNDING INFORMATION

This work was supported by funding from the University of Colorado Cancer Center Thoracic Oncology Research Initiative (to C.M.M and M.A.T.), the National Cancer Institute (R21CA252172 to R.B., C.M.M, and M.A.T.), and the NHLBI (5T32HL007085‐47 to R.B.). Biorender was used to create Figure 1a,b.

## CONFLICT OF INTEREST STATEMENT

C.M.M serves as the Vice Chair of the Board of Directors for the Colorado BioScience Institute. All other authors declare no potential conflicts of interest.

## ETHICS STATEMENT

Animal studies were reviewed and approved by the University of Colorado Anschutz Medical Campus Institutional Animal Care and Use Committee.

## Data Availability

Data supporting this study are available upon request to the corresponding author.
